# Straightening the Eyes Doesn't Rebalance the Brain

**DOI:** 10.3389/fnhum.2017.00453

**Published:** 2017-09-12

**Authors:** Jiawei Zhou, Yonghua Wang, Lixia Feng, Jiafeng Wang, Robert F. Hess

**Affiliations:** ^1^School of Ophthalmology and Optometry and Eye Hospital, and State Key Laboratory of Ophthalmology, Optometry and Vision Science, Wenzhou Medical University Wenzhou, China; ^2^Department of Ophthalmology, First Affiliated Hospital, Anhui Medical University Hefei, China; ^3^McGill Vision Research, Department of Ophthalmology, McGill University Montreal, QC, Canada

**Keywords:** sensory eye balance, contrast-gain-control, binocular vision, strabismic surgery, interocular suppression

## Abstract

Surgery to align the two eyes is commonly used in treating strabismus. However, the role of strabismic surgery on patients' binocular visual processing is not yet fully understood. In this study, we asked two questions: (1) Does realigning the eyes by strabismic surgery produce an immediate benefit to patients' sensory eye balance? (2) If not, is there a subsequent period of “alignment adaptation” akin to refractive adaptation where sensory benefits to binocular function accrue? Seventeen patients with strabismus (mean age: 17.06 ± 5.16 years old) participated in our experiment. All participants had normal or corrected to normal visual acuity (LogMAR < 0.10) in the two eyes. We quantitatively measured their sensory eye balance before and after surgery using a binocular phase combination paradigm. For the seven patients whose sensory eye balance was measured before surgery, we found no significant change [*t*_(6)_ = −0.92; *p* = 0.39] in the sensory eye balance measured 0.5–1 months after the surgery, indicating that the surgical re-alignment didn't by itself produce any immediate benefit for sensory eye balance. To answer the second question, we measured 16 patients' sensory eye balance at around 5–12 months after their eyes had been surgically re-aligned and compared this with our measurements 0.5–1 months after surgery. We found no significant change [*t*_(15)_ = −0.89; *p* = 0.39] in sensory eye balance 5–12 months after the surgery. These results suggest that strabismic surgery while being necessary is not itself sufficient for re-establishing balanced sensory eye dominance.

## Introduction

Ocular dominance, an eye preference in binocular viewing, commonly exists in humans (Chaurasia and Mathur, 1976). In clinical practice, ocular balance is normally determined by qualitative tests, e.g., the hole-in-the-card test (Dane and Dane, 2004), the Worth-4-dot test (Mustonen et al., 1993), which are convenient for clinical assessments, but not sufficient in providing a quantitative measure of the magnitude of asymmetry in binocular visual processing. In the recent decade, several studies have shown that the magnitude of asymmetry in different binocular visual processes, ranging from the binocular phase combination (Ding and Sperling, 2006; Huang et al., 2009), binocular orientation combination (Yehezkel et al., 2016), dichoptic motion coherence perception (Mansouri et al., 2008), dichoptic orientation coherence perception (Zhou et al., 2013a) to binocular rivalry (Ooi and He, 2001; Xu et al., 2011), can be quantitatively determined by the contrast difference of the visual inputs to achieve a binocular balanced status (i.e., the extent of binocular imbalance between the sensory inputs of each eye, or called “*sensory eye balance”*).

**Figure 1:**
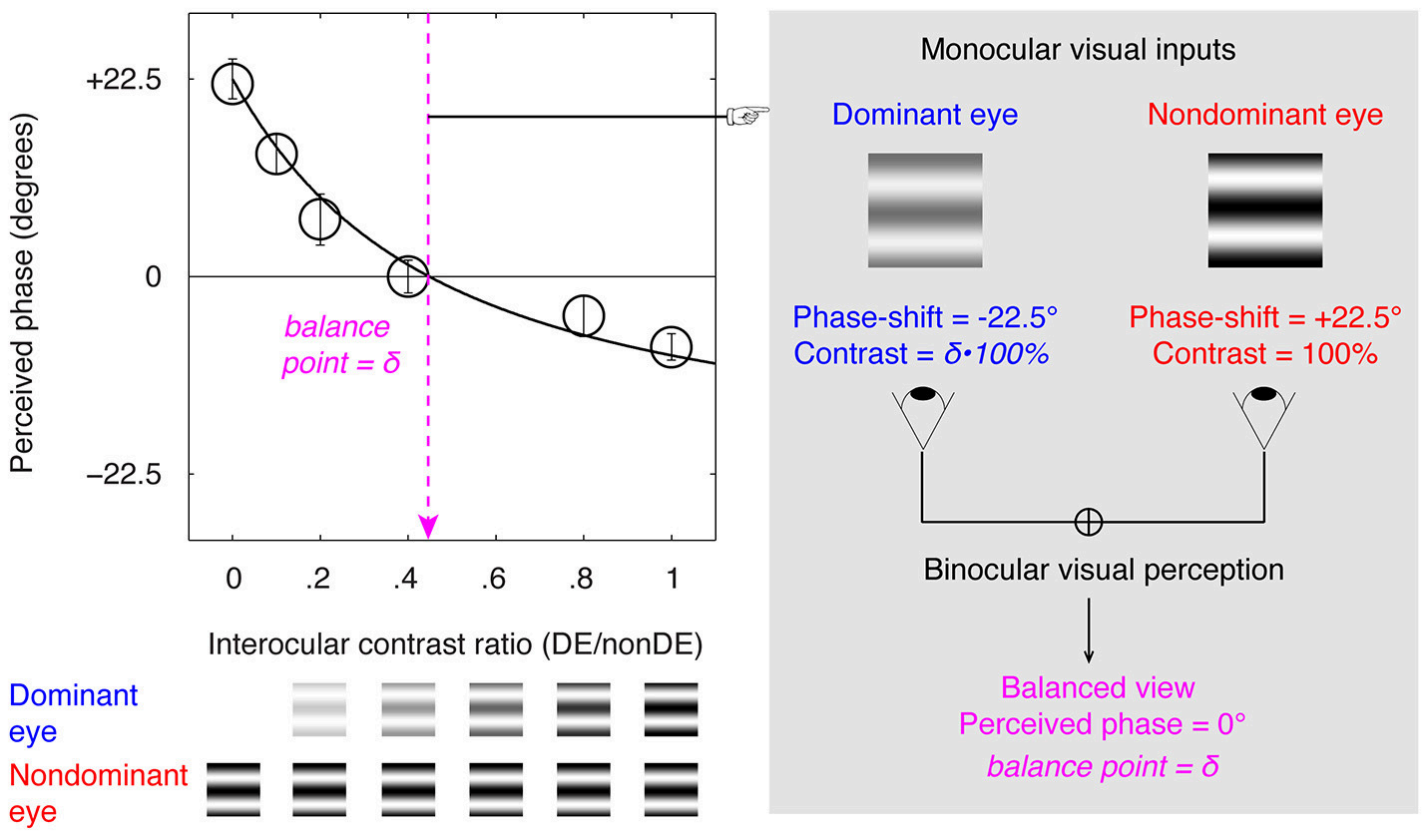
Illustration of the binocular phase combination paradigm. Two horizontal sine-wave gratings with equal and opposite phase-shifts of 22.5° (relative to the center of the screen) were dichoptically presented to the two eyes through the polarized glasses. The perceived phase of the cyclopean grating depended on the internal weights given to the two inputs. Sensory eye balance was quantified by the interocular contrast difference that was needed to achieve a 0-degree of perceived phase, i.e., the balance point, where the two eyes were balanced in binocular combination.

An illustration of the binocular phase combination task is provided in Figure 1, in which the two eyes are presented with two horizontal sine-wave gratings; the phase of the gratings in the two eyes are of same value but of opposite sign; the sensory eye balance is quantified by the interocular contrast ratio that is needed when the two eyes contribute equally to the binocular percept (i.e., when the binocular perceived phase is 0 degrees). Using this task, studies have shown that the sensory eye balance is abnormal in patients with amblyopia (Huang et al., 2009), anisometropia (Zhou et al., 2016), and strabismus (Kwon et al., 2014), which can be explained by a contrast-gain control theory in which the good eye suppresses the weak eye, thus unbalancing the monocular contributions to binocular processing (Ding and Sperling, 2006; Huang et al., 2010). Recently, we have also shown that the sensory eye balance remains abnormal in surgically corrected intermittent exotropes (Feng et al., 2015), indicating an abnormality of binocular processing in these patients. A related question that remains unanswered is, what is the effect of squint surgery on sensory eye balance? In other words, would straightening the eyes by itself lead to an improvement in sensory eye balance? Several previous studies have shown that in about 30–75% of cases binocularity and stereopsis are improved following successful surgical alignment, including adults with strabismus (Kushner and Morton, 1992; Morris et al., 1993; O'Neal et al., 1995; Yildirim et al., 1999; Lal and Holmes, 2002; Mets et al., 2004; Murray et al., 2007; Fatima et al., 2009; Dickmann et al., 2013). Sensory eye balance is an important binocular visual function and one would expect similar improvement to that referred to above for binocularity and stereopsis. However, there is a possibility that correcting the motor deficits may not be sufficient by itself to improve the binocular balance as reflected in the binocular phase combination task which has a cortical basis (Smith et al., 1997; Ding and Sperling, 2006; Huang et al., 2010). Besides, there is evidence that different binocular processes that share a similar interocular contrast-gain control stage may have separate pathways (Huang et al., 2010, 2011; Hou et al., 2013). It is possible that patients have deficits at different sites within the binocular pathway and this might explain inconsistencies in the effect of surgery on binocular function.

To test this, we measured the sensory eye balance using the binocular phase combination in a group of non-amblyopic, strabismic patients before, 0.5–1 months after and 5–12 months after strabismic surgery. We found no consistent short-term or long-term change in the binocular balance after the eyes had been straightened, suggesting that strabismic surgery while being necessary is not sufficient to reinstate this normal binocular function.

## Materials and methods

### Participants

Seventeen strabismic patients (mean age: 17.06 ± 5.16 years old) participated in our experiment. They were recruited from the department of Ophthalmology of the First Affiliated Hospital of Anhui Medical University (Anhui, China). All participants had normal or corrected to normal visual acuity (LogMAR < 0.10) in the two eyes. Some of the patients had anisometropia or myopia; their refractive errors had been corrected with eyeglasses at least 16 weeks before they participated in this study. None of the patients had amblyopia, a previous history of ocular surgery or strabismus surgery, sensory or paralytic exotropia, had nystagmus or other conditions limiting ocular movements, neurologic disorders or any other medical problems, or had undergone any kinds of vision training or patching treatment. Patients were excluded from the study if they were not successfully surgically corrected (a successful surgical alignment was defined as an exotropia of no more than 10 prism diopters at both far and near distance using the prism cover test) or any vertical misalignment. The left eyes of patients S3, S8, S9, and S17 and the right eyes of the other patients were strabismic. Clinical details of patients before and after surgery are provided in Table 1. Individuals' sensory eye balance was assessed before and after the surgery at different time points. Observers wore their prescribed optical correction, if needed, in the data collection.

**Table 1:** Clinical details of the participants.

**Subject#**	**Age (years old)/sex**	**Cycloplegic refractive errors (OD/OS)**	**LogMAR visual acuity (OD/OS)**	**Squint @ distance (D) and near (N), in prism diopters**		
				**Pre-surgery**	**0.5–1 months after surgery**	**5–12 months after surgery**
S1	10/M	Plano	0	XT60 XT′60	Ortho	Ortho
		Plano	0	XT60 XT′60	Ortho	Ortho
S2	13/F	−1.00DS	−0.10	X(T)55 X(T)′50	Ortho	Ortho
		−1.25DS	−0.10	X(T)55 X(T)′50	Ortho	Ortho
S3	19/F	−2.50DS	0.09	XT70 XT′70	X5 X′5	X5 X′7
		Plano	−0.10	XT70 XT′70	X5 X′5	X5 X′7
S4	15/F	−3.50DS	0.09	ET40 ET′40	Ortho	Ortho
		−3.25DS	0.09	ET40 ET′40	Ortho	Ortho
S5	14/M	−2.50DS	0.09	XT90 XT′90	X5 X′5	X8 X′8
		−3.00DS	0.09	XT90 XT′90	X5 X′5	X8 X′8
S6	19/F	−1.50DS	0	X(T)60 X(T)′60	E5 E′5	Ortho
		−3.50DS	0	X(T)60 X(T)′60	E5 E′5	Ortho
S7	14/M	Plano	0	XT75 XT′80	X3 X′3	Ortho
		Plano	0	XT75 XT′80	X3 X′3	Ortho
S8	16/F	−4.50DS	0	XT60 XT′65	Ortho	Ortho
		−4.75DS	0	XT60 XT′65	Ortho	Ortho
S9	28/F	−1.00DS/−0.75DC^*^75	0.09	X(T)50 X(T)′50	Ortho	Ortho
		Plano	0.09	X(T)55 X(T)′55	Ortho	Ortho
S10	16/F	−2.50DS	0	X(T)85 X(T)′90	Ortho	Ortho
		Plano	0	X(T)85 X(T)′90	Ortho	Ortho
S11	12/M	Plano	−0.20	XT80 XT′80	E10 E′10	E10 E′10
		Plano	−0.10	XT75 XT′75	E8 E′8	E8 E′8
S12	29/F	−7.25DS/−1.00DC^*^90	0	ET65 ET′65	E5 E′5	E6 E′6
		−7.00DS/−0.50DC^*^75	0	ET65 ET′65	E5 E′5	E6 E′6
S13	18/M	−4.00DS/−4.00DC^*^180	0.09	X(T)75 X(T)′75	X6 X′6	X10 X10
		−6.50DS/−2.50DC^*^180	0.09	X(T)75 X(T)′75	X6 X′6	X10 X10
S14	15/F	Plano	0	X(T)75 X(T)′70	Ortho	Ortho
		Plano	0	X(T)75 X(T)′70	Ortho	Ortho
S15	22/M	−3.25DS	0	XT85 XT′90	X5 X′5	X8 X′8
		−6.50DS	0	XT85 XT′90	X5 X′5	X8 X′8
S16	14/F	Plano	0.09	X(T)50 X(T)′50	Ortho	X4 X2
		−0.75DS	0.09	X(T)50 X(T)′50	Ortho	X4 X2
S17	16/M	−3.00DS	0	ET50 ET′50	Ortho	E6 E′6
		−3.00DS	0	ET50 ET′50	Ortho	E6 E′6

*F, Female; M, Male; OD, Oculus dexter (right eye); OS, Oculus sinister (left eye); DS, Dioptres sphere; DC, Dioptres cylinder; XT, Heterotropia Exodeviation at far distance (6 m); XT', Heterotropia Exodeviation at near distance (33 cm); X(T), Intermittent Exodeviation at far distance (6 m); X(T)', Intermittent Exodeviation at near distance (33 cm); ET, Heterotropia Esodeviation at far distance (6 m); ET', Heterotropia Esodeviation at near distance (33 cm); X, Heterophoria Exodeviation at far distance (6 m); X', Heterophoria Exodeviation at near distance (33 cm); E, Heterophoria Esodeviation at far distance (6 m); E', Heterophoria Esodeviation at near distance (33 cm)*.

All subjects were naive as to the purpose of the experiment. A written informed consent was obtained from each of them or from the parents or legal guardian of participants aged less than 18 years old, after explanation of the nature and possible consequences of the study. This study complied with the Declaration of Helsinki and was approved by the Institutional Review Boards of Wenzhou Medical University, Anhui Medical University and McGill University.

### Apparatus

All measurements were conducted on a PC computer running Matlab (MathWorks, Inc., Natick, MA) with PsychToolBox 3.0.9 extensions (Brainard, 1997; Pelli, 1997). The stimuli were presented on a gamma-corrected LG D2342PY 3D LED screen (LG Life Science, Korea) with a 1,920 × 1,080 resolution and a 60 Hz refresh rate. Subjects viewed the display dichoptically with polarized glasses in a dimly lit room at a viewing distance of 136 cm. The background luminance was 46.2 cd/m^2^ on the screen and 18.8 cd/m^2^ through the polarized glasses. A chin-forehead rest was used to minimize head movements during the experiment. Prisms (no more than 50 prism diopters) were added for some observers in the pre-surgery measurement to enable patients to align the two eyes. This was conducted, base-in for exotropia and base-out for esotropia, by fixing the prism on a trial frame using tape.

### Design

We quantitatively accessed patients' sensory eye balance before the surgery, 0.5–1 months after the surgery and 5–12 months after the surgery. Limited by the ability to align the two eyes, we were only able to measure the pre-surgery sensory eye balance in seven of the 17 patients. All patients were able to fuse the two eyes and thus were able to measure their sensory eye balance after the surgery.

A binocular phase combination paradigm (Ding and Sperling, 2006; Huang et al., 2009), which quantified the contributions of each eye to the fused binocular percept, was used for quantifying sensory eye balance. The design was the same as the one we have used in previous studies (Zhou et al., 2013a,b; Feng et al., 2015), in which observers were asked to dichoptically view two horizontal sine-wave gratings having equal and opposite phase-shifts of 22.5° (relative to the center of the screen) through polarized glasses; the perceived phase of the grating in the cyclopean percept was measured as a function of the interocular contrast ratio. By this method, we were able to find a specific interocular contrast ratio where the perceived phase of the cyclopean grating was 0° indicating equal weight to each eye's image. This specific interocular contrast ratio is the “balance point” for binocular phase combination since the two eyes under these stimulus conditions contribute equally to binocular vision (Figure 1). For each interocular contrast ratio, two configurations were used in the measurement so that any potential starting positional bias will be cancelled out: in one configuration, the phase-shift was +22.5° in the non-dominant eye and –22.5° in the dominant eye and in the other, the reverse. The perceived phase of the cyclopean grating at each interocular contrast ratio (δ) was quantified by half of the difference between the measured perceived phases in these two configurations. Different conditions (2 configurations × 6 interocular contrast ratios × 8 repetitions) were randomized in different trials, thus adaptation or expectation of the perceived phase would not have affected our results. The perceived phase and its standard error were calculated based on eight measurement repetitions.

Before the start of data collection, proper demonstration of the task was provided by practice trials to ensure observers understood the task. During the test, observers were allowed to take short-term breaks whenever they felt tired.

### Stimuli

The gratings in the two eyes, as shown in Figure 1, were defined as:

(1) $$\begin{array}{lll} Lum_{nonDE}\left(y\right) & = & L_{0}\left[1-C_{0}cos\;\left(2\pi fy\pm \frac{\theta }{2}\right)\right] \end{array}$$

(2) $$\begin{array}{lll} Lum_{DE}\left(y\right) & = & L_{0}\left[1-\delta C_{0}cos\;\left(2\pi fy\mp \frac{\theta }{2}\right)\right] \end{array}$$

Where *L*_0_ is the background luminance; *C*_0_ is the base contrast in the non-dominant eye; *f* is the spatial frequency of the gratings, δ is the interocular contrast ratio, and θ is the interocular phase difference.

In our test, *L*_0_ = 46.2 cd/m^2^ (on the screen); *C*_0_ = 100%; *f* = 1 cycle/°; δ = [0, 0.1, 0.2, 0.4, 0.8, 1.0] and θ = 45°.

The gratings had a size of 2° × 2°. Surrounding the gratings, a high-contrast frame (width, 0.11°; length, 6°) with four white diagonal lines (width, 0.11°; length, 2.83°) was always presented during the test to help observers maintain fusion.

### Procedure

We used the same phase adjustment procedure as used by Huang et al. (2009) for measuring the perceived phase of the binocularly combined grating. In each trial, subjects first completed an alignment task. During which, a fixation display was presented in the center of the binocular presented high-contrast frame with four white diagonal lines to the each eye. The fixation display also contained two monocular presented dots in the first and third quadrants for the left eye and two in the second and fourth quadrants for the right eye. They adjusted the coordinates of images in their nondominant eye to make sure the images seen by the two eyes were perfectly fused. This was followed by the binocular phase combination task. Observers were asked to adjust the position of a binocular horizontal reference line to indicate the perceived phase of the fused sine-wave grating. They did so by aligning the line with the location of the center of the dark stripe of the grating. The reference line was presented on both sides of the gratings, with its initial vertical position randomly (–9 to 10 pixels) assigned relative to the center of the frame in each trial. The reference line was moved with a fixed step size of 1 pixel. Since the gratings had a period of 2 cycles corresponding to 180 pixels, the phase adjustment had a step size of 4° of phase/pixel (2 cycles × 360 phase-degree/cycle / 180 pixels). During one trial, the stimuli were presented continually until subjects finished the phase adjustment task. The next trial started immediately after observers reported their results using a key press.

### Curve fits

The functions of perceived phase (φ) vs. interocular contrast ratios (δ) i.e., the PvR functions, were fitted with a modified contrast-gain control model from Huang et al. (2009):

(3) $$\varphi ={\text{tan}}^{-\text{1}}\left[\frac{1-{\left(\delta /bp\right)}^{1+\gamma }}{1+{\left(\delta /bp\right)}^{1+\gamma }}\;\cdot \text{ tan}\left(\frac{\theta }{2}\right)\right]$$

In which, *bp* and γ are two free parameters. “*bp*” represents the interocular contrast ratio when the two eyes make equally contributions to binocular combination (i.e., the balance point) and “γ” represents a non-linear factor.

Curve fitting was conducted in Matlab (MathWorks, Natick, MA) using the nonlinear least squares method to minimized Σ(φ_*theory*_–φ_*observed*_)^2^. The goodness-of-fit was statistically tested by computing the *r-square* value:

(4) $$\begin{array}{l} r^{2}=1-\frac{\sum {\left(\varphi_{theory}-\varphi_{observed}\right)}^{2}}{\sum {\left[\varphi_{observed}-mean\left(\varphi_{observed}\right)\right]}^{2}} \end{array}$$

## Results

### The immediate effect of strabismic surgery

**Figure 2:**
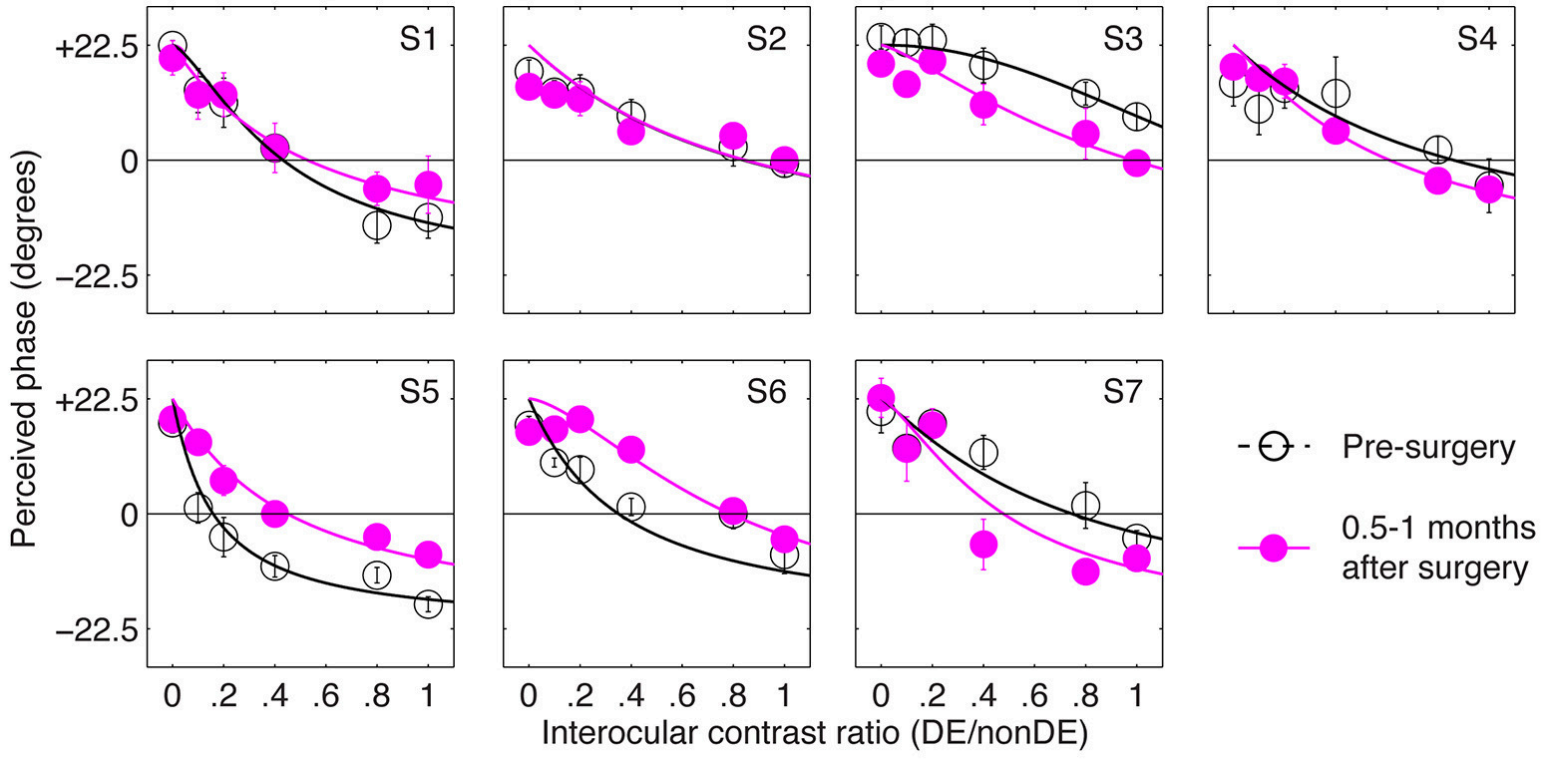
The immediate effect of strabismic surgery on patients' sensory eye balance. Binocular perceived phase was measured at different interocular contrast ratios (dominant eye/non-dominant eye) before and 0.5–1 months after surgery. Seven observers who were able to fuse the two eyes before surgery participated in the before surgery measurements. Error bars are standard errors.

Figure 2 shows the perceived phase vs. interocular contrast ratio (PvR) curves for seven patients, whose sensory eye balance could be measured before the surgery (other patients were not measureable because they couldn't align the two eyes during the test). For observer S3, her nondominant eye (determined by the hole-in-the-card test) was more dominant in the pre-surgery PvR test, to simplify the statistical analysis, S3's balance points were calculated as the reciprocal of the fitted balance point in the comparison. Accordingly, all the seven patients had imbalanced eyes before the surgery, as evidenced by the significant distance between the balance points (the zero-crossing point of the PVR curves) and 1.0 (i.e., the ideal observers' balance point): *t*_(6)_ = –4.03, *p* = 0.007. At 0.5–1 months after the surgery, patients' eyes were more imbalanced (balance point decreased by 0.2 or more: S4 and S7), less imbalanced (balance point increased by 0.2 or more: S3, S5, and S6) or remained imbalanced to the same extent (balance point changed by less than 0.2: S1 and S2).

**Figure 3:**
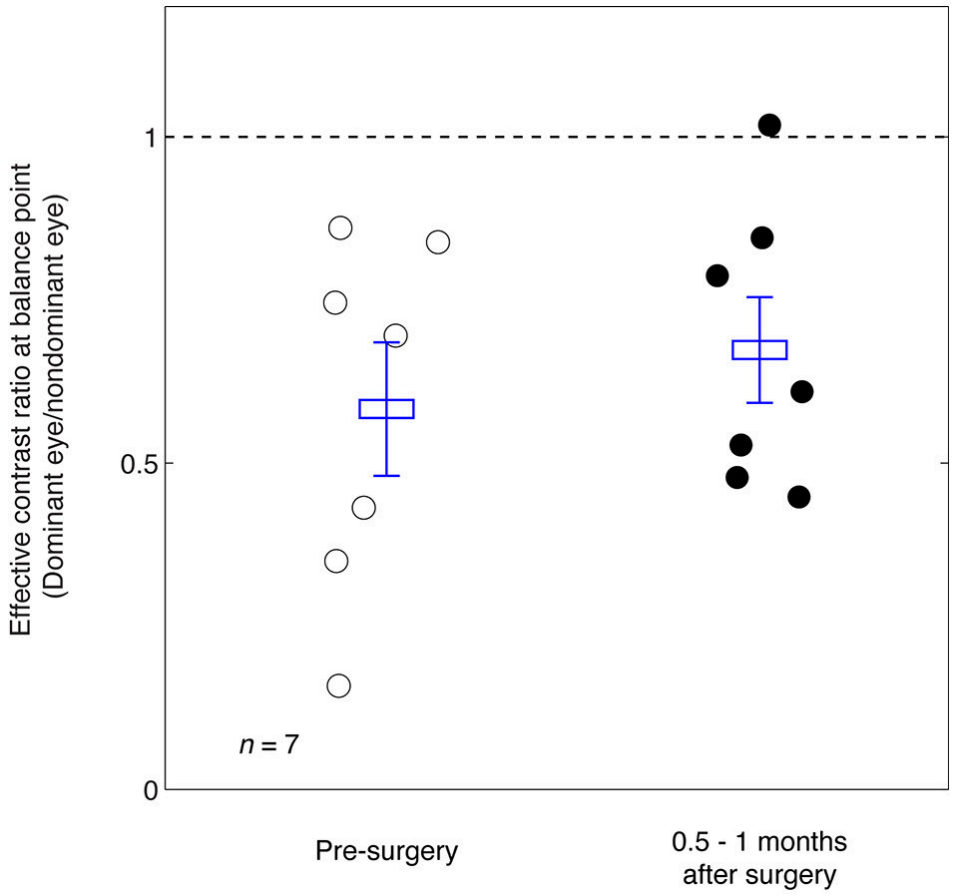
Summary of the balance point changes before and immediately after strabismic surgery. The mean and standard errors are indicated and suggest the surgery does not in itself contribute to a more balanced ocular dominance.

In Figure 3 we plot the balance points derived from the results shown in Figure 2 for the seven observers whose balance was measured before and immediately after strabismic surgery. The means and standard error are illustrated. A 2-tailed paired samples *t*-test showed that there was no significant change in the balance point as a consequence of surgery: *t*_(6)_ = –0.92, *p* = 0.39.

### The long-term effect of strabismic surgery—“surgical adaptation”

**Figure 4:**
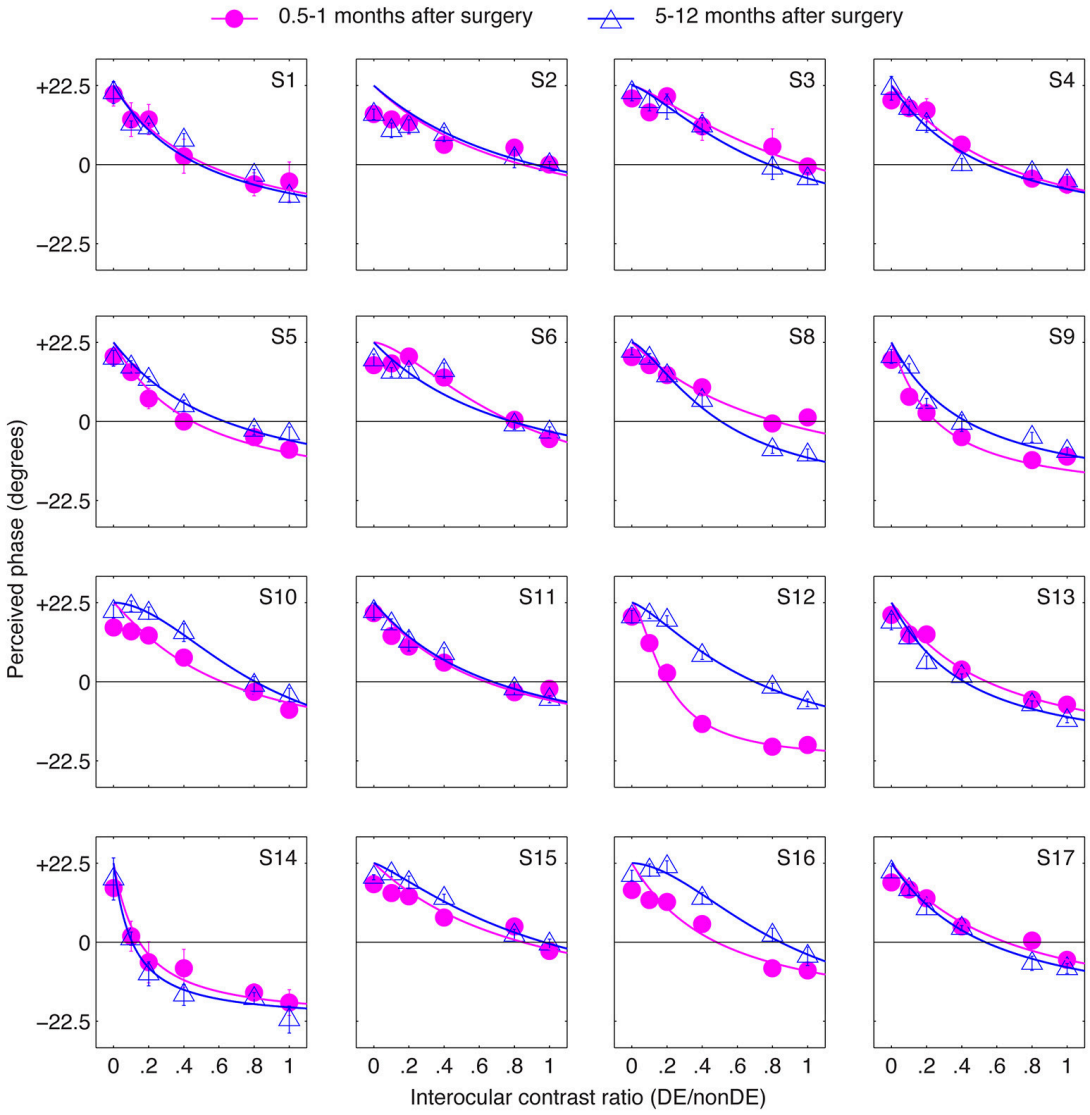
The long-term effect of strabismic surgery. Binocular perceived phase was measured at different interocular contrast ratios (dominant eye/non-dominant eye) at 5–12 months after surgery and compared to that at 0.5–1 months after surgery. Sixteen observers participated. Error bars are standard errors.

The results in Figure 3 suggest that strabismic surgery doesn't produce any immediate benefit in terms of patients' sensory eye balance. One possibility is that a period of post-surgical “adaptation,” somewhat akin to refractive adaptation after an interocular refractive imbalance is first corrected (Zhou et al., 2016). To test this possibility, we evaluated the long-term effect of strabismic surgery on patients' sensory eye balance by measuring patients' PvR functions at 5–12 months after surgery. These results are plotted in Figure 4. Except for observers S12 and S16, whose balance point increased by more than 0.2 (two eyes became more balanced) at 5–12 months after the surgery, all other observers' balance points generally remained unaltered (balance point changed by less than 0.2) by surgery.

**Figure 5:**
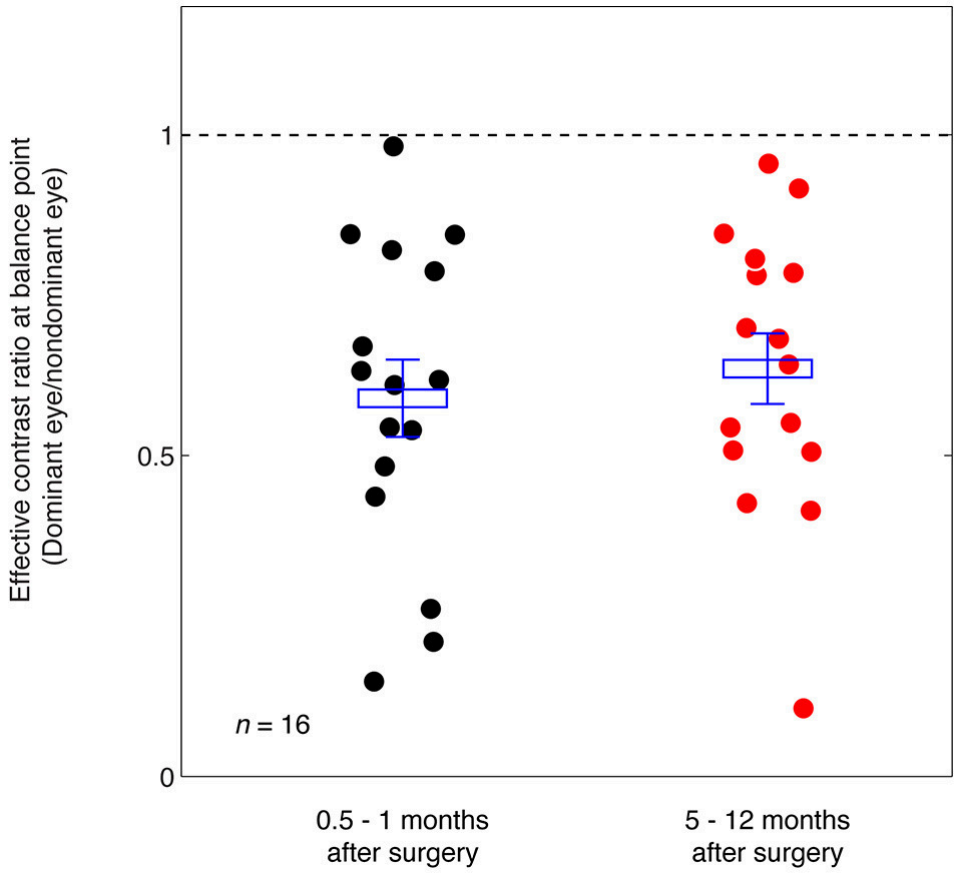
Summary of the balance point changes immediately after (0.5–1 months) strabismic surgery compared with at 5–12 months after the surgery. The mean and standard errors are indicated and suggest the surgery does not in itself contribute to a more balanced ocular dominance.

Figure 5 summarizes the range of balance points we measured in our strabismic patients immediately after surgery and how they changed 5–12 months after surgical straighting of the eyes. A 2-tailed paired samples *t*-test also showed that there was no significantly change of balance point after 5–12 months: *t*_(15)_ = −0.89, *p* = 0.39. There was also no significant correlation between the interval between the two post-operation measure days and the change of balance point (*p* = 0.24, 2-tailed Pearson Correlation).

## Discussion

We have shown previously (Zhou et al., 2016) that anisometropes have imbalanced ocular dominance as a consequence of an uncorrected refractive difference between the eyes and that this binocular balance anomaly, although unaffected by the initial optical correction, is nevertheless improved by a period of long-term refractive correction. We speculate that this may be the foundation for what is referred to as “refractive adaptation” in anisometropic amblyopia where visual acuity can improve solely as a consequence of long-term spectacle wear. In the present study we wanted to know whether there was a similar beneficial adaptation (i.e., surgical adaptation) for balanced binocular function after correction of an interocular eye misalignment. We quantified eye balance by measuring the contribution that each eye made to the binocularly fused percept using a standard psychophysical approach that is based on a well-accepted neural model of binocular combination (Ding and Sperling, 2006; Meese et al., 2006). By varying the interocular contrast we derived balance point measures that reflect the eye dominance. The results indicated that while strabismics could exhibit a range of eye dominances, the mean eye dominance for a group of observers did not change immediately (0.5–1 months) after surgery, nor did it change 5–12 months after surgery. In other words, we found no benefit of the surgery *per se* on binocular eye balance in the short or the long term, hence no evidence for “surgical adaptation” when it comes of eye balance.

Surgical re-alignment is clearly a first step in providing the conditions necessary for re-establishing full binocular function in strabismus. It has been shown that there can be benefits to binocularity including reduced suppression, better fusion and improvements in stereopsis in a significant percentage of cases (Kushner and Morton, 1992; Morris et al., 1993; O'Neal et al., 1995; Yildirim et al., 1999; Lal and Holmes, 2002; Mets et al., 2004; Murray et al., 2007; Fatima et al., 2009; Dickmann et al., 2013). However, surgical alignment *per se* does not seem to initiate any long-term changes in terms of rebalancing eye dominance in cases of strabismics without amblyopia. Thus, while some improvements can occur in a minority of cases (as detailed above) presumably even with a disorder ocular balance, we hypothesize that improvements will be more substantial and more frequent when any ocular dominance imbalance is first corrected. To test this it will be necessary for future studies to restore normal ocular balance after surgical correction by using active training approaches along the lines as that used in cases of amblyopia (Hess et al., 2010; To et al., 2011; Ooi et al., 2013; Li et al., 2014, 2015) and assess whether this leads to better binocular outcomes.

## Author contributions

JZ, LF, and RH designed research; LF and JW performed research; JZ and YW analyzed data; JZ and RH wrote the paper. All authors revised the paper and approved the version to be published.

### Conflict of interest statement

The authors declare that the research was conducted in the absence of any commercial or financial relationships that could be construed as a potential conflict of interest.
